# Vocal individuality of Holstein-Friesian cattle is maintained across putatively positive and negative farming contexts

**DOI:** 10.1038/s41598-019-54968-4

**Published:** 2019-12-05

**Authors:** Alexandra Green, Cameron Clark, Livio Favaro, Sabrina Lomax, David Reby

**Affiliations:** 1 Livestock Production and Welfare Group, School of Life and Environmental Sciences, University of Sydney, Camden, Australia; 2 Equipe Neuro-Ethologie Sensorielle, ENES/CRNL, CNRS UMR5292, INSERM UMR_S 1028, University of Lyon/Saint-Étienne, Saint-Étienne, France; 3 Department of Life Sciences and Systems Biology, University of Turin, Via Accademia Albertina 13, 10123 Turin, Italy

**Keywords:** Animal behaviour, Biological techniques

## Abstract

Cattle mother-offspring contact calls encode individual-identity information; however, it is unknown whether cattle are able to maintain individuality when vocalising to familiar conspecifics over other positively and negatively valenced farming contexts. Accordingly, we recorded 333 high-frequency vocalisations from 13 Holstein-Friesian heifers during oestrus and anticipation of feed (putatively positive), as well as denied feed access and upon both physical and physical & visual isolation from conspecifics (putatively negative). We measured 21 source-related and nonlinear vocal parameters and stepwise discriminant function analyses (DFA) were performed. Calls were divided into positive (n = 170) and negative valence (n = 163) with each valence acting as a ‘training set’ to classify calls in the oppositely valenced ‘test set’. Furthermore, MANOVAs were conducted to determine which vocal parameters were implicated in individual distinctiveness. Within the putatively positive ‘training set’, the cross-validated DFA correctly classified 68.2% of the putatively positive calls and 52.1% of the putatively negative calls to the correct individual, respectively. Within the putatively negative ‘training set’, the cross-validated DFA correctly assigned 60.1% of putatively negative calls and 49.4% of putatively positive calls to the correct individual, respectively. All DFAs exceeded chance expectations indicating that vocal individuality of high-frequency calls is maintained across putatively positive and negative valence, with all vocal parameters except subharmonics responsible for this individual distinctiveness. This study shows that cattle vocal individuality of high-frequency calls is stable across different emotionally loaded farming contexts. Individual distinctiveness is likely to attract social support from conspecifics, and knowledge of these individuality cues could assist farmers in detecting individual cattle for welfare or production purposes.

## Introduction

Domesticated cattle are highly gregarious, residing in herds in both natural and commercial farming environments^1^. Within these herds, interactions over short and long distances are mediated by vocalisations^2^. Cattle produce two broad call types which are modulated by the configuration of the supra-laryngeal vocal tract^2^; including low-frequency nasalised calls for close contact and/or lower distress, and orally emitted high-frequency calls for distant communication and/or times of higher arousal^2,3^. In commercial farming environments, cattle are exposed to numerous procedures in which they emit these high-frequency vocalisations, but knowledge of their information content is limited. Calls are reported during oestrus^4,5^, separation from calf^6^, isolation from conspecifics^7^ and in anticipation of feed^8^, and likely encode information about the sender including their identity and emotional state^3,9,10^. Within the herd, advertising individuality in high-frequency calls would be biologically advantageous, by helping to facilitate social support from conspecifics. Moreover, recognising individual cattle could assist farmers in the non-invasive detection of welfare. However to date, these potential uses of cattle vocalisations have only been explored in cattle mother-offspring dyads, where low and high-frequency vocalisations were emitted to facilitate social interactions in a relatively undisturbed environment^2,11^.

Vocal cues to individuality are increasingly being found in wild^12–14^ and domesticated^2,15–17^ ungulate species, in relation to the source-filter theory^18^. According to the source-filter theory, vocalisations are produced by two independent processes, firstly with the sound generated by vibrations in the vocal folds (the source), and secondly with the sound filtered by the vocal tract (the filter)^18,19^. In ungulates, individuality is encoded in a range of source-related vocal parameters, including the F0-contour^12,16^, amplitude contour^15^ and duration^16^, as well as filter-related vocal parameters including formant frequencies^2,12^. Individuality expression seems to differ for each call type^2,20^ and in some ungulate species individuality has been shown to be more strongly expressed in oralised than nasalised calls^12,20^. In the context of mother-offspring contact, individual differences of cattle high-frequency calls were attributed to formants, but classification to the correct individual was relatively low^2^ suggesting that these calls were not very individualised. While formants are well established vocal indicators of individuality as they are influenced by caller morphology^21^, they can be poorly represented in high-pitch vocalisations^22,23^. Considering that cattle can produce vocalisations with fundamental frequencies over 1000 Hz^24^, which are likely to occur during times of higher arousal^3^, then vocal parameters unrelated to vocal tract resonances may better encode individuality information in high-frequency calls. It has been hypothesised that high-frequency calls should contain more individuality information than their low-frequency equivalents due to their propagation over longer distances where vision of the signaller is not always guaranteed^25^. Considering this evidence, cattle high-frequency calls emitted in stressful farming situations should indeed be highly individualised. On this basis we decided to study source-related, and nonlinear parameters in cattle high-frequency calls, as they have been shown to aid with individual identification in other species^26–29^.

As consecutive calls and calls of the same context are likely to be homogeneous^30,31^, they would likely result in high individual discriminability, regardless of whether they are truly idiosyncratic. To more robustly measure vocal individuality, studies should instead determine whether vocal individuality is maintained across time^14^, a variety of contexts and/or call types^32,33^. Thus, the aim of our study was to determine whether vocal individuality of cattle high-frequency calls is maintained across positively and negatively valenced contexts. This is of particular interest considering that different emotional experiences can influence the sound of the voice^3,33^. We hypothesised that individual distinctiveness is encoded in the max F0 as well as percentages of nonlinear phenomena in each call, and indeed that vocal individuality would be maintained across valences.

## Results

### Vocal individuality

Descriptive statistics for all the measured vocal parameters from individual heifers during the putatively positive and negative valenced contexts are provided in the Supplementary Material (Tables S1–S2). The MANOVA revealed significant differences between the 13 heifers in the acoustic structure of their high-frequency putatively positive calls (Pillai’s Trace, F_252, 1776_ = 4.787, P < 0.001), as well as their putatively negative calls (Pillai’s Trace, F_252, 1692_ = 4.289, P < 0.001). In the putatively positive calls, separate univariate ANOVAs revealed that the individual effect of heifer was significant for all acoustic variables (all P < 0.002) except for subharmonics (P = 0.240). Similar results were obtained from the putatively negative calls, where separate univariate ANOVAs again revealed a significant individual effect of heifer for all acoustic variables (all P < 0.02) except for subharmonics (P = 0.590).

Discriminant function analyses (DFA) indicated that heifers maintain vocal individuality across putatively positive and negative contexts. Using the 170 putatively positive calls as a ‘training set’, the DFA produced eight statistically significant discriminant functions, which were used to classify 78.2% of the putatively positive calls to the correct heifer. This DFA classification slightly decreased to 68.2% when the more conservative leave-one-out cross-validation procedure was undertaken. Upon using the putatively positive calls as a ‘training set’, 52.1% of the putatively negative calls were classified to the correct individual in the ‘test set’. Using the 163 putatively negative calls as a ‘training set’, the DFA produced six statistically significant discriminant functions, which were used to classify 70.6% of the putatively negative calls to the correct heifer. The classification of the DFA slightly decreased to 60.1% when the more conservative leave-one-out cross-validation procedure was undertaken. Additionally, the putatively negative ‘training set’ allowed for the classification of 49.4% of the putatively positive calls to the correct individual in the ‘test set’.

**Figure 1:**
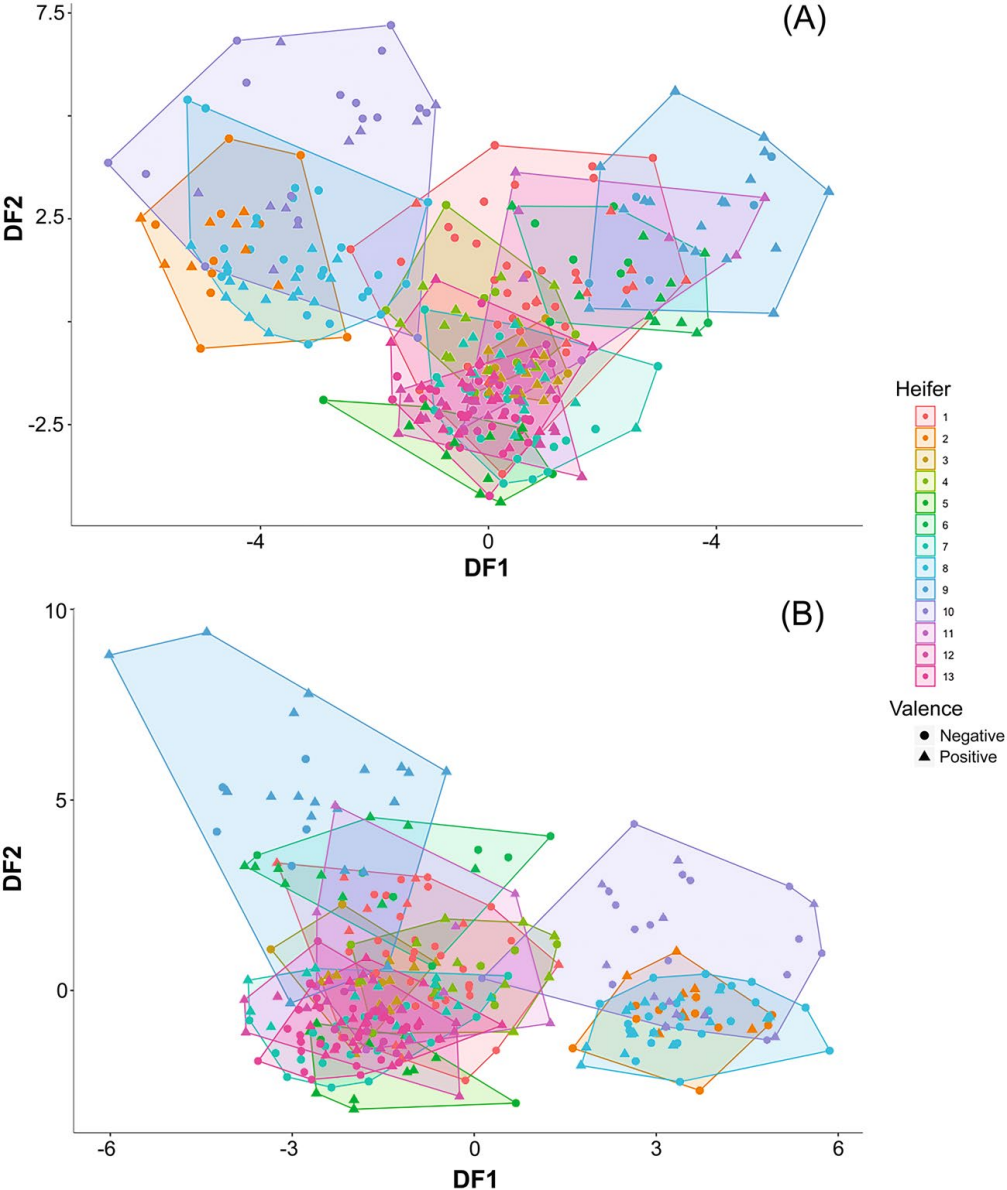
Discriminant function scores for all calls derived from the putatively positively valenced ‘training set’ (**A**) and the putatively negatively valenced ‘training set’ (**B**), illustrating vocal individuality between the 13 heifers across both positive and negative valence.

The two-tailed binomial tests confirmed that all the DFAs were significantly above chance expectation (all: n = 13, group size chance expectation = 7.69%, P < 0.001). The statistical tests for the canonical discriminant functions are provided in the Supplementary Material (Table S3). The stepwise procedures were performed in 10 and nine steps for the putatively positive and negative ‘training sets’ respectively (Table 1). In the putatively positive ‘training set’, the first two discriminant functions revealed five vocal parameters that highly contributed to individual distinctiveness, including AM var, AM rate, F0 max, F0 var and biphonation sidebands %. In the putatively negative ‘training set’, six vocal parameters attributed to individual distinctiveness including AM var, AM rate, F0 max, F0 var, harmonicity and duration. Figure 1 displays the vocal distinctiveness of individuals across discriminant function scores one and two.

**Table 1:** Standardised canonical discriminant function coefficients obtained by the stepwise procedure to classify vocalisations to the correct heifer using the putatively positive ‘training set’ and putatively negative ‘training set’. Bold values relate to factor loadings > ± 0.5.

	Vocal parameter	Discriminant function									
		1	2	3	4	5	6	7	8	9	10
(Putatively positive training set)	Entropy	0.361	0.415	0.233	**0**.**921**	**0**.**556**	0.321	−0.083	**0**.**703**	−0.013	−0.077
	Harmonicity	−0.003	0.418	0.428	**0**.**815**	−0.013	0.388	**0**.**857**	**1**.**089**	0.355	0.137
	AM var	−**0**.**867**	**0**.**647**	−0.163	−0.012	0.119	−0.227	−0.230	0.190	0.247	−0.255
	AM rate	−**0**.**670**	0.463	0.009	0.170	0.177	0.399	0.432	−0.026	−0.014	**0**.**702**
	F0 max	**0**.**707**	**1**.**119**	0.276	−**1**.**198**	**0**.**615**	−0.146	0.248	0.131	0.076	−0.421
	F0 start	0.412	0.326	0.386	−0.086	−0.040	0.240	−0.416	−0.190	**0**.**776**	−0.046
	F0 var	−0.072	−**0**.**851**	−**0**.**642**	**1**.**561**	−**0**.**755**	−0.410	0.195	−**0**.**591**	−0.171	**0**.**690**
	Jitter	0.240	−0.268	−0.451	−0.167	0.309	**0**.**604**	0.421	**0**.**923**	0.495	−0.066
	Biphonation sidebands	0.035	**0**.**562**	0.188	−0.096	−0.251	**0**.**582**	0.039	−0.238	−0.401	−**0**.**635**
	Frequency jumps	0.097	0.165	−0.211	−0.009	−**0**.**624**	0.136	−**0**.**574**	0.405	−0.064	0.376
(Putatively negative training set)	Entropy	0.269	0.415	**1**.**180**	0.299	0.007	−0.353	−0.231	−0.132	−0.065	
	Harmonicity	**0**.**817**	0.250	**1**.**208**	**0**.**928**	0.247	**0**.**657**	−0.151	**0**.**736**	0.059	
	AM var	**1**.**313**	−0.187	−0.005	−0.409	0.047	0.076	−0.245	−**0**.**589**	0.201	
	AM rate	**0**.**711**	0.176	−0.341	0.437	0.013	−0.367	0.234	**0**.**518**	−0.334	
	F0 max	−0.452	**1**.**806**	−**0**.**529**	−**0**.**992**	−**0**.**831**	−0.199	−**1**.**011**	0.430	−0.374	
	F0 min	−0.182	0.489	−0.201	0.402	−0.034	−0.138	−0.047	−0.274	**0**.**764**	
	F0 var	0.100	−**0**.**991**	0.246	**1**.**115**	**1**.**234**	**0**.**507**	**1**.**614**	−**0**.**664**	0.063	
	Shimmer	0.423	−0.202	**0**.**926**	−0.226	0.422	0.251	−0.157	**0**.**927**	**0**.**727**	
	Duration	**0**.**774**	−0.443	0.181	−0.447	−0.325	0.238	0.804	−0.643	0.760

## Discussion

We investigated whether high-frequency vocalisations of cattle emitted during putatively positive and negative contexts encoded information on individuality. We showed that a range of vocal parameters are implicated in individual distinctiveness in cattle high-frequency calls. Moreover, results revealed that vocal individuality is stable across putatively positive and negative contexts. This is the first study to show that cattle maintain vocal identity cues across a variety of farming situations. Our results contribute to the understanding of cattle vocal communication and have the potential to assist with the non-invasive assessment of cattle welfare.

Due to their function in long-distance communication and more frequent production during highly arousing contexts^3^, we expected that the high-frequency calls would contain salient cues to individuality. Accordingly, the discriminant function analyses revealed that cattle high-frequency calls are individually distinct. In the cross-validated DFA, high-frequency cattle calls were assigned to the correct individual at least 60% of the time within the same emotional valence and at least 49% across emotional valences, with all classifications exceeding chance expectations. Moreover, our DFA classification percentages both within and across valence were higher than previously reported in cattle high-frequency contact calls, where the cross-validated DFA resulted in 30.9% classification, and formant parameters were mostly responsible for individual identity^2^. While high-frequency calls contribute less in mother-offspring recognition^2,11^, our results suggest that during other farming contexts, vocal cues to individuality play an important role in the recognition of familiar conspecifics.

Through interpreting the high factor loadings of the first two discriminant functions, the DFA indicated that a combination of vocal parameters was implicated in individual distinctiveness of cattle high-frequency calls. Across the putatively positive and negative datasets, the DFA consistently relied on AM var, AM rate, F0 var and F0 max to build the discriminant functions. The MANOVA then confirmed this vocal individuality, with all the parameters selected for this study, bar subharmonics, significantly differing between heifers. While many vocal individuality studies have examined formant frequencies due to their relationship with caller morphology^2,14,21^, this was less practical in the present study where calls were characterised by very high F0s and consequently less clear formant frequencies^22,23^. Nevertheless, our results reveal that a range of source and nonlinear parameters have the potential to encode individuality information, which has similarly been demonstrated in the groans of fallow deer, where formants were less important cues to individuality^29^.

In the present study, to minimise over-estimation of individual classification percentages, we classified data that was not used to build the original discriminant functions^34^. Our study utilised two separate datasets for DFA training and testing, with data including inhomogeneous samples from five farming contexts emitted over different days. This novel and robust approach enabled an increase in confidence around the accuracy of the analysis and subsequent stability of vocal individuality. We found that classification performance slightly declined when using the putatively positive and negative ‘training sets’ to classify vocal individuality in their respective opposite putatively positive or negative ‘test set’. Nonetheless, classification values across ‘test sets’ remained significantly higher than chance levels emphasising that vocal individuality is maintained across putatively positive and negative contexts. Likewise, vocal cues to identity have been found to remain stable in kittens exposed to different emotionally arousing situations^35^ and deer over the rutting period^14^, suggesting that the salience of vocal individuality during different emotionally loaded contexts is indeed biologically advantageous. In both the putatively positive and negative calls, the combination of high F0 and abundance of nonlinear phenomena is a likely consequence of high subglottal pressure^23,36^, reflecting the high-arousal that the heifers were experiencing^3^ in both the putatively positively valenced oestrus and anticipation of feed, and the putatively negatively valenced isolation and feed denial. For cattle, which are a social herd-living prey-species^1^, emitting idiosyncratic calls, especially during times of high arousal, could facilitate altruism directed from conspecifics^37^, with whom they develop stable social relationships^1^.

In the current dataset, the slight decline in classification percentages across valences could be explained by two reasons. Firstly, due to the rarity of vocalisations emitted by heifers in some contexts and the difficulty in obtaining high-quality vocalisations in commercial farming environments, the calls were unevenly distributed across putatively positive and negative valence. Secondly, this classification decline could relate to the within-heifer vocal variability which likely arose from cattle being exposed to different emotionally loaded contexts. Changes in emotional state have been shown to result in modulations of vocalisations^3,31,33,38^, with a growing body of literature on vocal indicators of emotion in pigs^39,40^, horses^38,41^ and goats^31,42^. Thus, in addition to identity cues, vocal cues of emotion should be studied in cattle high-frequency calls.

We demonstrated that cattle that vocalise during positive situations should, in theory, be able to be recognised by conspecifics when they vocalise in a negative situation, and vice versa. Although vocalisations were produced under different emotional contexts, they shared cues to identity. We selected calls of the extreme high-frequency call type as these were the most commonly produced across the five recording contexts. However, if we interpret cattle vocalisations as being produced on a graded continuum of low to high-frequency^43^, based on emotional arousal and/or underlying motivation^3^, then it would be interesting to determine whether individuality is also maintained across the entire cattle vocal repertoire of low to high-frequency calls. The ability of heifers to recognise individuals based on vocal individuality cues both within and across different emotionally valenced contexts should also be confirmed using playback experiments. Since calves can recognise their mother’s low-frequency calls during mother-offspring communication^11^, it is highly likely that cows too can recognise their familiar conspecifics within the herd using their high-frequency calls. Anecdotally, farmers have also described being able to distinguish between their cattle using only their vocalisations. To confirm this, we also recommend further studies conducting psychoacoustics experiments to determine which vocal cues of individuality farmers attend to in cattle high-frequency calls. This knowledge could help farmers in identifying individual cattle requiring welfare intervention.

## Conclusion

In conclusion, we demonstrated that a variety of source and nonlinear-related vocal parameters are responsible for encoding individuality in Holstein-Friesian heifer high-frequency calls. Further, by using robust classification methods, we showed that heifers can maintain this individual distinctiveness across putatively positive and negative farming contexts. We suggest that salience to individuality in cattle high-frequency calls assists with the recognition of familiar conspecifics in the herd. We recommend that farmers integrate knowledge of these cues into their daily farming practices for cattle welfare or production improvements.

## Materials and Methods

### Study site and animals

This experiment was undertaken in a free-ranging environment at the University of Sydney, Australia, “Wolverton Farm” between June and October 2017. A herd of 18 Holstein-Friesian non-pregnant virgin heifers were recorded for this experiment. Heifers were selected to be uniform in breed, production status, age (24.5 ± 2.5 months) and weight (412.8 ± 44.7 kg) to control for their excessive influence on vocal individuality. The heifers were situated in a 4 Ha paddock containing cattle yards, where they had access to native pasture, unlimited water and were supplemented with lucerne hay (dry matter: 89.1%, crude protein: 16.4%, metabolisable energy: 8.5%) daily.

The heifers were recorded producing high-frequency open-mouth vocalisations during oestrus, two feeding contexts and two isolation contexts. Prior to the commencement of recording, heifers were adapted to the presence of human observers (between two and four people concurrently), as well as the routine of moving through the cattle yards for sorting and husbandry procedures. To assist with the identification of individual heifers during the recording contexts, heifers were assigned numbers which were spray-painted with fluoro stock-mark on either side of their flank. Spray painting was conducted with the heifers restrained in a head-bail and cattle crush in the cattle yards. Low-stress handling methods were always implemented when moving the heifers to and from the paddock and cattle yards. All procedures were approved by the University of Sydney animal ethics committee ‘IRMA’ (project number: 2016/1078), with the recording contexts only causing temporary distress to the heifers involved. All procedures were performed in accordance with the Australian code for the care and use of animals for scientific purposes^44^.

### Audio recordings and contexts

Vocalisations were recorded using a Sennheiser K6-ME67 directional microphone (frequency response: 40 to 20000 Hz, max SPL: 125 dB at 1 kHz, Sennheiser Electronic, Wedemark, Germany) attached to a Marantz PMD-661 MK2 digital solid-state recorder (Marantz Professional, United Kingdom). The microphone was directed towards the vocalising heifer as best as possible. For shock and wind-noise reduction, the microphone was protected with a Rycote Classic Softie Windshield ®. Further, recordings were only taken when weather was permissible. Each vocal recording was saved as a separate file in the.WAV uncompressed format at 44.1 kHz sampling rate and 16-bit amplitude resolution. Vocal recordings were obtained when the same cattle were: 1) in oestrus, 2) anticipating feed, 3) denied feed access, 4) physically isolated from conspecifics, and 5) physically and visually isolated from conspecifics. Recordings were carried out during daylight hours between 08:00 and 17:00 with no recordings collected later than 17:00 h due to sound interference from the cattle feeding tractors and limited daylight. Specific details about the recording contexts are provided in the Supplementary Methods.

### Inferences about emotional valence in the recording contexts

The recording contexts were classified as positive or negative, according to their putative emotional valence. We did not need precision with emotional valence classification in the present study, since we were determining whether vocal individuality could be maintained across contexts and time. Therefore we inferred emotional valence of the oestrus, feeding and isolation contexts based on the functions of emotions^45–48^, and knowledge of livestock behaviour^31,38,49^. Positive emotions are part of the pleasant-appetitive motivational system, which trigger approach towards releasing stimuli, while negative emotions are part of the unpleasant-defensive motivational system, which trigger avoidance of releasing stimuli^45–48^. Subsequently, oestrus was assumed to be positively valenced, as during this time cattle exhibited affiliative behaviours including approaching conspecifics, sexual behaviours including anogenital sniffing and licking, and exploratory behaviours in search of a mate^47,48^. At the ultimate level, oestrus functions to promote survival, allowing for the attraction of a mate and potential procreation^45^. Anticipation of feed was also deemed to be positively valenced since feeding should induce approach behaviour and increase fitness in the wild. Contrastingly, both physical and physical and visual social isolation were assumed to be negatively valenced, since cattle are highly gregarious and being separated from the herd could threaten fitness. Further, denial of feed access was assumed to be negatively valenced, as it could lead to frustration, lack of feed intake in the wild and an overall threat to fitness^31,46^. While all 18 heifers were exposed to the five recording contexts, not all heifers vocalised within each context. Nonetheless, we obtained vocalisations in at least one of the positive and one of the negative recording contexts for each heifer (Table 2).

**Table 2:** Number of calls analysed from each heifer including the contexts and putative valences in which they were produced.

Positive valence				Negative valence			
Heifer ID	Oestrus	Anticipation of feed	Total	Physical isolation	Physical and visual isolation	Feed denial	Total
1	0	10	10	10	10	10	30
2	1	7	8	0	0	10	10
3	3	10	13	0	0	3	3
4	3	9	12	3	0	4	7
5	10	0	10	0	0	2	2
6	0	10	10	0	0	7	7
7	5	10	15	9	2	10	21
8	8	8	16	10	10	4	24
9	10	9	19	1	0	4	5
10	1	9	10	4	2	10	16
11	6	1	7	0	0	2	2
12	10	10	20	0	0	10	10
13	10	10	20	10	6	10	26

### Vocalisation selection

Cattle vocalisations are classified into two broad types, namely low-frequency and high-frequency calls which are modulated by configuration of the supra-laryngeal vocal tract^2,24^. During the oestrus recording context, low-frequency closed-mouth calls were seldom observed. For our acoustic analyses, we therefore only focused on the high-frequency open-mouth calls, as these were directly comparable in the heifers across the putatively positive and negative farming contexts. Calls were selected based on their high signal to noise ratio and in the absence of wind or signal saturation, resulting in a total of 333 calls analysed from 13 of the 18 heifers (Table 2). Despite the low incidence of calling from some individuals, calls were also balanced as much as possible across the putative valences. Additionally, if calls were produced as part of a sequence, we only selected them for analyses if they were more than 10 s apart in order to reduce homogeneity associated with consecutive calling. In total, 53 of the 333 vocalisations were derived from sequences of low and high-frequency vocalisations, with only two vocalisations selected from the same sequence considering they were non-consecutive.

### Vocalisation analyses

**Figure 2:**
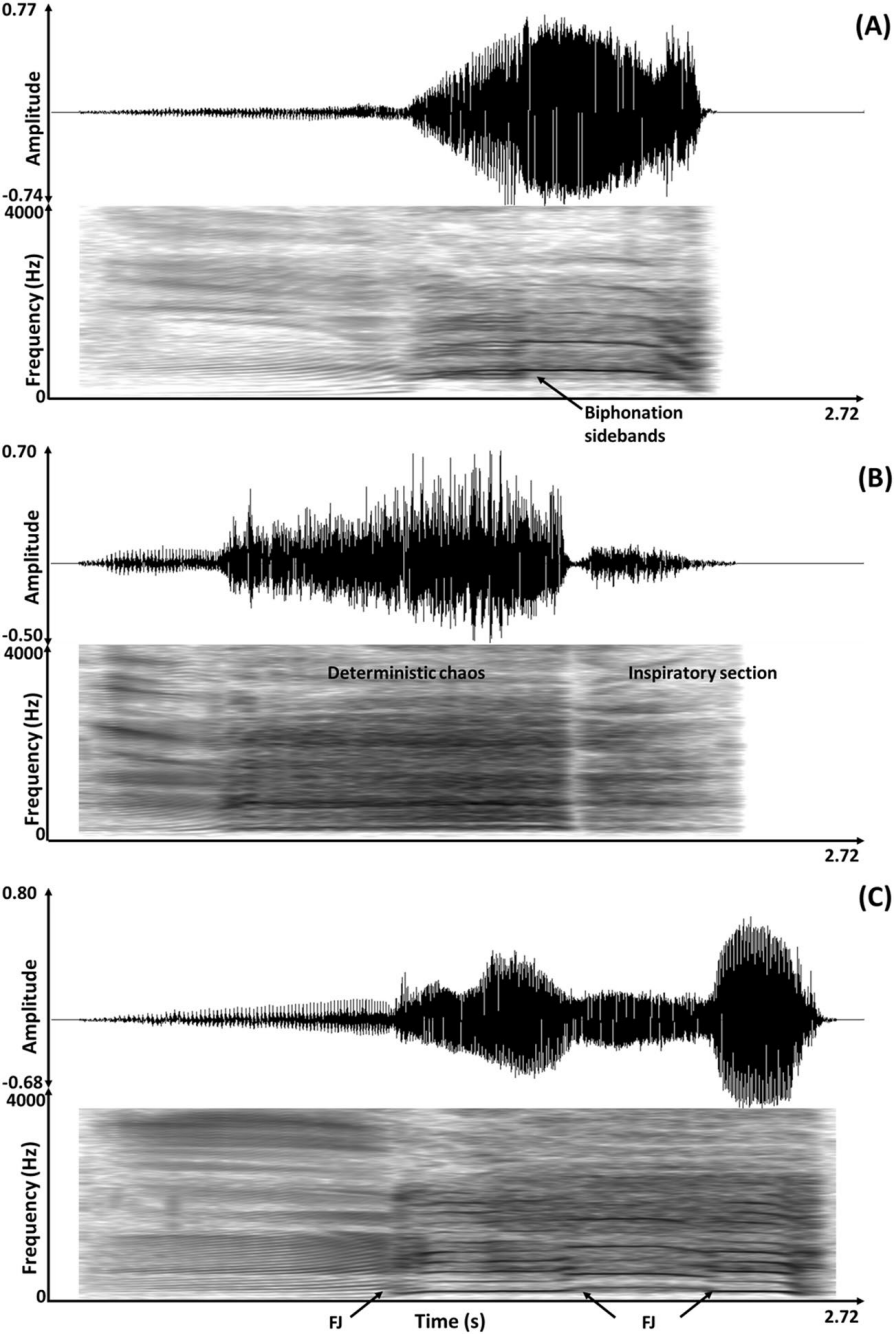
Sample oscillograms (top) and narrow-band spectrograms (bottom) of vocalisations recorded during the putatively positive and negative contexts from three different heifers, containing nonlinear phenomena including (**A**) biphonation sidebands during anticipation of feed, (**B**) deterministic chaos during denial of feed and (**C**) frequency jumps (FJ) during oestrus. Spectrograms were visualised in Praat v.6.0.31 (FFT method, window length = 0.1 s, time steps = 1000, frequency steps = 250, Gaussian window shape, dynamic range = 60 dB).

Vocalisations were analysed using Praat DSP package v.6.0.31^50^, through both calculation off the oscillograms and spectrograms; and by using a series of custom-built scripts^31,51^ to automatically extract a range of acoustic features. Vocalisations were visualised as narrow-band spectrograms (FFT method, window length = 0.1 s, time steps = 1000, frequency steps = 250, Gaussian window shape, dynamic range = 60 dB) and a total of 21 vocal parameters were measured in each of the vocalisations (Table 3). Prior to running the scripts, the full duration (s) of the call was measured directly off the oscillogram. Nonlinear phenomena were widely prevalent in the calls including 80% and 93% of the putatively positive and negative calls, respectively (See Supplementary Methods for further details on prevalence). For this reason, the percentages of nonlinear phenomena relative to the full call duration were calculated off the spectrogram. Nonlinear phenomena criteria were adopted from previous vocal studies in non-human mammalian species^52–55^ and included deterministic chaos, biphonation sidebands, subharmonics, and frequency jumps. Example waveforms and spectrograms of the nonlinear phenomena are provided in Fig. 2.

**Table 3:** Description of the 21 vocal parameters measured for each vocalisation.

Parameter type	Vocal parameter	Definition
Temporal	Duration (s)	Total duration of the call (from start to end)
Spectral	Entropy	Quantification of signal randomness ranging from 0 which is a pure tone to 1 which is random noise
Frequency	F0 mean (Hz)	Mean F0 frequency across the call
	F0 min (Hz)	Minimum F0 frequency across the call
	F0 max (Hz)	Maximum F0 frequency across the call
	F0 start (Hz)	F0 frequency at the start of the call
	F0 end (Hz)	F0 frequency at the end of the call
Frequency modulation	Inflex 2	Index of strong F0 variation
	F0 var (Hz/s)	Cumulative variation in the F0 contour in Hertz divided by call duration
	FM rate (s^−1^)	Number of complete cycles of frequency modulation per second
	FM extent (dB)	Mean peak to peak variation of each frequency modulation
Periodicity	Harmonicity (dB)	Harmonic to noise ratio of the call
	Jitter (%)	Cycle to cycle frequency variation in the F0
	Shimmer (%)	Cycle to cycle amplitude variation in the F0
Intensity	AM var (Hz/s)	Cumulative variation in the amplitude divided by call duration
	AM rate (s^−1^)	Number of complete cycles of amplitude modulation per second
	AM extent (dB)	Mean peak to peak variation of each amplitude modulation
Nonlinear phenomena	Deterministic chaos (%)	Non-random broadband noise with no clear harmonic structure
	Subharmonics (%)	Integer fractions of the F0
	Biphonation sidebands (%)	Side frequencies which occur either side of the F0 and harmonics due to amplitude modulation
	Frequency jumps (frequency)	Abrupt and discontinuous changes to the F0, often separated by a period of silence, which occur in upwards or downwards directions

Before extracting the vocal parameters, a script was run to add silences of 0.1 s to each side of the 333 calls. Using custom-built scripts in Praat^31,51^, we then batch-processed the acoustic analyses, with output data exported to Microsoft Excel for further examination. In the script, pitch floor and ceiling settings were adapted to the individual heifer voices and these settings were maintained across calls collected during positive and negative valence for a given heifer. Specific Praat procedures are detailed in the Supplementary Methods.

### Statistical analyses

Statistical analyses were performed using SPSS v.24 (IBM Corp. Released 2016). Two separate stepwise discriminant function analysis (DFA) procedures were used to quantify the extent of which individual heifers could be classified based on their calls. The DFAs were conducted both within each putative valence and across the putative positive and negative valences to establish whether individual differences in the high-frequency calls of heifers are maintained. In both DFAs, the grouping variable was heifer (1–13), the discriminant variables were the 21 vocal parameters and the selection variable was valence (positive = 1 or negative = 2). A first DFA was run with the selection variable set to 1 (positive). In this DFA, the 170 putatively positive calls were used as a ‘training set’, to firstly classify the 170 putatively positive calls to the correct individual, and secondly classify the 163 putatively negative calls in the ‘test set’ to the correct individual. Then, a second DFA was run with the selection variable set to 2 (negative). In this DFA the 163 putatively negative calls were used as a ‘training set’ to firstly classify the 163 putatively negative calls to the correct individual, and secondly classify the 170 putatively positive calls in a ‘test set’ to the correct individual. For both DFAs, we used the default settings for the F value of the model, which included an entry level of 3.84 and a removal level of 2.71. Since there was an imbalance in the number of vocalisations from each heifer across positive and negative valence, the percentage of correct classification was calculated according to the group sizes. We used the leave-one-out classification procedure to cross-validate the results and the Wilks’ lambda method to determine how strongly each of the discriminant functions contributed to the models. To confirm the accuracy of the DFA classifications, we used two-tail binomial tests to see whether correct classifications were significantly higher than chance expectations^34,35,56^. Graphical representations of the first two discriminant functions scores for heifer vocal individuality were additionally formulated in R Studio v.1.1.463 using the ggplot2 package^57^.

We also conducted two multivariate general linear models (MANOVA) using the putatively positive and negative calls separately, to determine whether there were significant differences between heifers in their 21 vocal parameters. In both MANOVAs, heifer was included as the categorical fixed factor and the 21 vocal parameters were used as the independent variables. Descriptive statistics (means ± SE) are provided in the Supplementary Materials for all vocal parameters of heifers during putative positive and negative valence.

## Supplementary information

Supplementary materials

## References

[1] Rault JL (2012). Friends with benefits: Social support and its relevance for farm animal welfare. Appl. Anim. Behav. Sci..

[2] Padilla de la Torre M, Briefer EF, Reader T, McElligott AG (2015). Acoustic analysis of cattle (Bos taurus) mother–offspring contact calls from a source–filter theory perspective. Appl. Anim. Behav. Sci..

[3] Briefer EF (2012). Vocal expression of emotions in mammals: Mechanisms of production and evidence. J. Zool..

[4] Schön PC (2007). Altered vocalization rate during the estrous cycle in dairy cattle. J. Dairy Sci..

[5] Röttgen Volker, Becker Frank, Tuchscherer Armin, Wrenzycki Christine, Düpjan Sandra, Schön Peter C., Puppe Birger (2018). Vocalization as an indicator of estrus climax in Holstein heifers during natural estrus and superovulation. Journal of Dairy Science.

[6] Weary DM, Chua B (2000). Effects of early separation on the dairy cow and calf. Appl. Anim. Behav. Sci..

[7] Boissy A, Le Neindre P (1997). Behavioral, Cardiac and Cortisol Responses to Brief Peer Separation and Reunion in Cattle. Physiol. Behav..

[8] Yeon SC (2006). Acoustic features of vocalizations of Korean native cows (Bos taurus coreanea) in two different conditions. Appl. Anim. Behav. Sci..

[9] Green A. C., Johnston I. N., Clark C. E. F. (2017). Invited review: The evolution of cattle bioacoustics and application for advanced dairy systems. animal.

[10] Watts JM, Stookey JM (2000). Vocal behaviour in cattle: The animal’s commentary on its biological processes and welfare. Appl. Anim. Behav. Sci..

[11] Padilla de la Torre M, Briefer EF, Ochocki BM, McElligott AG, Reader T (2016). Mother–offspring recognition via contact calls in cattle, Bos taurus. Anim. Behav..

[12] Volodin IA (2017). Individuality of distress and discomfort calls in neonates with bass voices: Wild-living goitred gazelles (Gazella subgutturosa) and saiga antelopes (Saiga tatarica). Ethology.

[13] Reby D, Joachim J, Lauga J, Lek S, Aulagnier S (1998). Individuality in the groans of fallow deer (Dama dama) bucks. J. Zool..

[14] Reby D, André-Obrecht R, Galinier A, Farinas J, Cargnelutti B (2006). Cepstral coefficients and hidden Markov models reveal idiosyncratic voice characteristics in red deer (Cervus elaphus) stags. J. Acoust. Soc. Am..

[15] Sèbe F, Poindron P, Ligout S, Sèbe O, Aubin T (2017). Amplitude modulation is a major marker of individual signature in lamb bleats. Bioacoustics.

[16] Blackshaw JK, Jones DN, Thomas FJ (1996). Vocal individuality during suckling in the intensively housed domestic pig. Appl. Anim. Behav. Sci..

[17] Favaro L, Briefer BF, McElligott AG (2014). Artificial neural network approach for revealing individuality, group membership and age information in goat kid contact calls. Acta Acust. United with Acust..

[18] Taylor AM, Reby D (2010). The contribution of source-filter theory to mammal vocal communication research. J. Zool..

[19] Titze, I. R. *Principles of voice production*. (Prentice-Hall Inc., 1994).

[20] Volodin IA, Lapshina EN, Volodina EV, Frey R, Soldatova NV (2011). Nasal and Oral Calls in Juvenile Goitred Gazelles (Gazella subgutturosa) and their Potential to Encode Sex and Identity. Ethology.

[21] Taylor, A. M., Charlton, B. D. & Reby, D. Vocal production by terrestrial mammals: Source, filter and function. In *Vertebrate Sound Production and Acoustic Communication* 241–259 (2016).

[22] Erickson ML, D’Alfonso AE (2002). A comparison of two methods of formant frequency estimation for high-pitched voices. J. Voice.

[23] Raine J, Pisanski K, Bond R, Simner J, Reby D (2019). Human roars communicate upper-body strength more effectively than do screams or aggressive and distressed speech. PLoS One.

[24] Volodin IA, Volodina EV, Frey R (2017). Bull bellows and bugles: a remarkable convergence of low and high-frequency vocalizations between male domestic cattle Bos taurus and the rutting calls of Siberian and North American wapiti. Bioacoustics.

[25] Leliveld LMC, Scheumann M, Zimmermann E (2011). Acoustic correlates of individuality in the vocal repertoire of a nocturnal primate (Microcebus murinus). J. Acoust. Soc. Am..

[26] Digby A (2014). Non-linear phenomena in little spotted kiwi calls. Bioacoustics.

[27] Fitch WT, Neubauer J, Herzel H (2002). Calls out of chaos: the adaptive significance of nonlinear phenomena in mammalian vocal production. Anim. Behav..

[28] Zhang Fang, Zhao Juan, Feng Albert S. (2017). Vocalizations of female frogs contain nonlinear characteristics and individual signatures. PLOS ONE.

[29] Vannoni E, McElligott AG (2007). Individual acoustic variation in fallow deer (Dama dama) common and harsh groans: A source-filter theory perspective. Ethology.

[30] Briefer E, McElligott AG (2011). Indicators of age, body size and sex in goat kid calls revealed using the source-filter theory. Appl. Anim. Behav. Sci..

[31] Briefer EF, Tettamanti F, McElligott AG (2015). Emotions in goats: mapping physiological, behavioural and vocal profiles. Anim. Behav..

[32] Elie, J. E. & Theunissen, F. E. Zebra finches identify individuals using vocal signatures unique to each call type. *Nat*. *Commun*. **9** (2018). 10.1038/s41467-018-06394-9 PMC6168511 30279497

[33] Lavan Nadine, Burton A. Mike, Scott Sophie K., McGettigan Carolyn (2018). Flexible voices: Identity perception from variable vocal signals. Psychonomic Bulletin & Review.

[34] Mundry R, Sommer C (2007). Discriminant function analysis with nonindependent data: consequences and an alternative. Anim. Behav..

[35] Scheumann M (2012). Vocal correlates of sender-identity and arousal in the isolation calls of domestic kitten (Felis silvestris catus). Front. Zool..

[36] Suthers Roderick A., Fitch W. Tecumseh, Fay Richard R., Popper Arthur N. (2016). Vertebrate Sound Production and Acoustic Communication.

[37] Tibbetts EA, Dale J (2007). Individual recognition: it is good to be different. Trends Ecol. Evol..

[38] Briefer EF (2015). Segregation of information about emotional arousal and valence in horse whinnies. Sci. Rep..

[39] Maigrot A, Hillmann E, Briefer E (2018). Encoding of Emotional Valence in Wild Boar (Sus scrofa) Calls. Animals.

[40] Friel M, Kunc HP, Griffin K, Asher L, Collins LM (2019). Positive and negative contexts predict duration of pig vocalisations. Sci. Rep..

[41] Maigrot AL, Hillmann E, Anne C, Briefer EF (2017). Vocal expression of emotional valence in Przewalski’s horses (Equus przewalskii). Sci. Rep..

[42] Baciadonna L, Briefer EF, Favaro L, McElligott AG (2019). Goats distinguish between positive and negative emotion-linked vocalisations. Front. Zool..

[43] Kiley M (1972). The Vocalizations of Ungulates, their Causation and Function. Z. Tierpsychol..

[44] National Health and Medical Research Council. *Australian code for the care and use of animals for scientific purposes - 8th Edition*. (2013).

[45] Bradley MM, Codispoti M, Cuthbert BN, Lang PJ (2001). Emotion and Motivation I: Defensive and Appetitive Reactions in Picture Processing. Emotion.

[46] Mendl M, Burman OHP, Paul ES (2010). An integrative and functional framework for the study of animal emotion and mood. Proc. Biol. Sci..

[47] Mellor DJ (2015). Positive animal welfare states and encouraging environment-focused and animal-to-animal interactive behaviours. N. Z. Vet. J..

[48] Mellor DJ (2012). Animal emotions, behaviour and the promotion of positive welfare states. N. Z. Vet. J..

[49] Leliveld LMC, Düpjan S, Tuchscherer A, Puppe B (2016). Behavioural and physiological measures indicate subtle variations in the emotional valence of young pigs. Physiol. Behav..

[50] Boersma, P. & Weenink, D. Praat: doing phonetics by computer, http://www.praat.org/ (2009).

[51] Reby D, McComb K (2003). Anatomical constraints generate honesty: acoustic cues to age and weight in the roars of red deer stags. Anim. Behav..

[52] Charlton BD (2015). The acoustic structure and information content of female koala vocal signals. PLoS One.

[53] Stoeger AS, Baotic A, Li D, Charlton BD (2012). Acoustic Features Indicate Arousal in Infant Giant Panda Vocalisations. Ethology.

[54] Stoeger AS (2011). Vocal cues indicate level of arousal in infant African elephant roars. Acoust. Soc. Am..

[55] Charlton, B. D., Martin-Wintle, M. S., Owen, M. A., Zhang, H. & Swaisgood, R. R. Vocal behaviour predicts mating success in giant pandas. *R*. *Soc*. *Open Sci*. **5** (2018). 10.1098/rsos.181323 PMC6227945 30473861

[56] Townsend SW, Charlton BD, Manser MB (2014). Acoustic cues to identity and predator context in meerkat barks. Anim. Behav..

[57] Wickham, H. ggplot2: Elegant Graphics for Data Analysis (2016).

