# Supplementary material for: Vocal individuality of Holstein-Friesian cattle is maintained across putatively positive and negative farming contexts
Source: Sci Rep. 2019 Dec 5;9:18468. doi: 10.1038/s41598-019-54968-4 (PMC6895157; doi:10.1038/s41598-019-54968-4)
Supplement: Supplementary file 1 — Supplementary materials [file 41598_2019_54968_MOESM1_ESM.pdf]

Vocal individuality of Holstein-Friesian cattle is maintained across putatively positive and negative farming contexts

Alexandra Green[1,2], Cameron Clark[1], Livio Favaro[2,3], Sabrina Lomax[1], David Reby[2]

[1]Livestock Production and Welfare Group, School of Life and Environmental Sciences, University of Sydney, Camden, Australia

[2]Equipe Neuro-Ethologie Sensorielle, ENES/CNRL, CNRS UMR5292, INSERM UMR_S 1028, University of Lyon/Saint-Étienne, Saint-Étienne, France

[3]Department of Life Sciences and Systems Biology, University of Turin, Via Accademia Albertina 13, 10123 Turin, Italy

Supplementary methods

Details of the acoustic recording contexts.

Over 60 days, the Holstein-Friesian heifers were subjected to five recording contexts where they were likely to vocalise. These included 1) oestrus, 2) feed-anticipation, 3) feed-denial, 4) physical isolation and, 5) physical and visual isolation. Recording was conducted in both a free-ranging environment and in a set of cattle yards. There was high inter-heifer vocal variability with 5 of the 18 heifers not vocalising enough or at all to be included in the vocalisation analyses. For Heifers 3, 5 and 11, only two or three high-frequency calls were obtained in the negative contexts across the entire duration of recording. Specific details about the recording contexts are provided below.

1) Oestrus was synchronised in the 18 heifers via intramuscular administration of PGF2α (Estrumate®, Cloprostenol sodium, Schering Plough Animal Health). Oestrus recordings commenced 48 h after the PGF2α administration and ceased approximately 96 h after the PGF2α administration. No oestrus-related activity was detected outside of this recording frame. Recordings were conducted thrice daily at 0900, 1200 and 1500, with each session lasting 90 minutes. Oestrus was confirmed at the beginning of each recording session by an experienced animal handler, through visual displays such as a reddened vulva, mucus production, increased activity and mounting. Additionally, Estrotect ™ self-adhesive patches were attached on the backbone of each heifer, between the hip and tail head to assist with the detection of mounting behaviour. The onset of oestrus varied between individual heifers. At 48 h post-pgf2α administration, six heifers had displayed oestrus activity, by 72 h another eight had displayed oestrus activity and by 96 h, the final four heifers had displayed oestrus activity. During oestrus, vocal production was closely accompanied by affiliative social contact (approaching conspecifics, allogrooming, standing to be mounted, licking or sniffing the anogenital region), as well as exploration of the paddock. Within the oestrus recording sessions, 15 of the 18 heifers vocalised. No bulls were present on the farm during oestrus cycling.

2 and 3) Heifers were recorded during anticipation of a feed-reward over ten sessions, followed by denial of feed access over another ten sessions. Each recording session lasted five minutes and occurred on separate days. The heifers were recorded in their paddock in groups rather than singularly, so we could reproduce the feeding routines that regularly occur on commercial farms. The heifers were recorded in groups of six, rather than in their whole herd of 18, to reduce poor-quality recordings that could result if the entire herd vocalised concurrently. Accordingly, calls from the feeding contexts were only selected if they were not overlapping. The groups of six heifers were kept consistent across all feed recordings, to reduce potential conflict that could occur between heifers introduced to new social groupings. When not involved in the feeding procedures, heifers were situated in the cattle yards.
The procedure of feed-anticipation involved a herdsperson, wearing a fluorescent yellow vest, with whom the heifers had previously associated feeding-time, carting an empty wheelbarrow to a hay bale, filling it with a portion of lucerne hay (approximately 1kg per heifer) and slowly transporting it back to the heifers. The herdsperson very obviously presented the feed to the heifers by breaking it up into small portions and shaking the wheelbarrow. After five minutes of feed presentation, the heifers were then offered the feed as reward. The heifers were allowed five minutes to finish eating before they were herded back to the cattle yards. The heifers were most vocal when the herdsperson reached the hay bale and on return with the wheelbarrow full of hay. During anticipation of feed, 14 of the 18 heifers were vocal. The procedure of feed-denial involved the same conspicuously dressed herdsperson, feeding one group of six heifers, namely 'fed heifers', whilst another group of six heifers, namely 'denied heifers' watched on in an adjacent paddock. The 'fed heifers' were continuously fed a portion of lucerne hay (approximately 1kg per heifer) for five minutes, over which time the herdsperson allowed the 'denied heifers' to see, lick and sniff the hay before it was instead provided to the 'fed heifers'. To maintain the display of heifer frustration during feed-denial, and to prevent the extinction of vocal responses over repeated sessions, after the five-minutes ceased, the denied heifers were either randomly offered feed, or returned to the cattle yards without a feed reward. During feed-denial, 14 of the 18 heifers were vocal.

4 and 5) In the cattle yards, heifers were exposed to four sessions of physical isolation from conspecifics, and four sessions of physical and visual isolation from conspecifics. Both isolation treatments were tested concurrently, with the order of isolation treatment alternated between heifers: half of the heifers were randomly assigned to physical isolation first, and the other half were randomly assigned to physical and visual isolation first. Each isolation session lasted 30 minutes, with up to eight heifers isolated per day in a randomised order. After completing the four sessions in their respective isolation treatment, heifers underwent a three-month washout period where no isolation testing was conducted. Heifers were then crossed over to their new respective isolation treatment and the methods were repeated. During physical isolation, heifers had full visual and auditory contact with their familiar conspecifics, but during physical and visual isolation, their vision of conspecifics was obstructed through the attachment of black opaque material to the cattle yard panels. In both isolation contexts, the heifer's familiar conspecifics were situated 50 m away in the paddock. One heifer was excluded from both social isolation contexts as she was overly stressed, resulting in 17 heifers participating in the isolation experiments. During physical isolation, 8 of the 17 heifers vocalised, whereas during physical and visual isolation only 5 of the 17 heifers vocalised.

Prevalence of different nonlinear phenomena types in the cattle vocalisations during putatively positive and negative valence.

During the putatively positive contexts, 80% of the vocalisations contained at least one type of nonlinear phenomena. Of the 170 putatively positive calls, 62% contained deterministic chaos, 15% contained subharmonics, 16% contained biphonation sidebands and 48% contained upward or downward frequency jumps. During the putatively negative contexts, 93% of the vocalisations contained at least one type of nonlinear phenomena. Of the 163 putatively negative calls, 74% contained deterministic chaos, 18% contained subharmonics, 29% contained biphonation sidebands and 61% contained upward or downward frequency jumps.

Praat methods are detailed below with commands parenthesised.

We measured a range of F0-related parameters derived from the F0 contour of each call using the cross-correlation method ([Sound: To pitch (cc) command], time step = 0.01 s, pitch floor = 40 – 60 Hz, pitch ceiling = 350 – 1000 Hz). We lowered the voicing threshold in the analysis to 0.25 to assist in tracking the F0 in calls where background noise was present. Spurious values and octave jumps were manually corrected by viewing the spectrogram and F0 contour in the Pitch edit window [Inspect pitch object][1]. When deterministic chaos was present, we 'unvoiced' incorrect F0 values and for 19/333 calls (n = 2 heifers), F0 was not measured across the entire call due to atonality. To characterise the F0, we measured the F0 mean, F0 min, F0 max, F0 start and F0 end over the duration of the call. Additionally, to characterise the F0 modulation along the call, we measured the F0 var, FM rate and FM extent. We then applied a smoothing algorithm (Smooth command in Praat, bandwidth = 2) to the F0 contour, to suppress the short-term frequency modulation[2,3]. With this algorithm, the number of inflection points was divided across the total duration of voiced segments in the call, resulting in a distinct index of F0 modulation, namely inflex 2, which provided good indication of major intonation events[2,3].

We then measured a range of intensity-related parameters derived from the intensity contour of each call [Sound: To intensity command]. These included AM var, AM rate and AM extent. To assess the periodic quality of the call, we also calculated the jitter [(Jitter (local) command)] and shimmer [(Shimmer (local) command)] using the same settings as per the F0 contour. Further, we calculated the harmonicity using the standard settings in Praat ([To harmonicity (cc)], time step = 0.01 s, pitch floor = 40 – 60 Hz, silence threshold = 0.1, periods per window = 1). Finally, we measured the entropy of the calls using the 'seewave package'[4] in R studio (v 3.5.2. R Development Core Team, 2018) using the standard settings (wl = 512, envt = "hil", msmooth = NULL, ksmooth = NULL).

References

1. Reby, D. & McComb, K. Anatomical constraints generate honesty: acoustic cues to age and weight in the roars of red deer stags. *Anim. Behav.* **65**, 519–530 (2003).

2. Raine, J., Pisanski, K., Simner, J. & Reby, D. Vocal communication of simulated pain. *Bioacoustics* **4622**, 1–23 (2018).

3. Koutseff, A. *et al.* The acoustic space of pain: cries as indicators of distress recovering dynamics in pre-verbal infants. *Bioacoustics* **4622**, 1–13 (2017).

4. Sueur, J. *et al.* Sound Analysis and Synthesis, Package 'seewave'. 1–201 (2018).

Supplementary materials

Table S1. Descriptive statistics (mean ± SE) for each of the 21 vocal parameters of a given heifer during the putatively positive contexts (n = 170).

**Putatively positively valenced Vocal parameter**	**Heifer** 1 (n = 10)	**2 (n = 8)**	**3 (n = 13)**	**4 (n = 12)**	**5 (n = 10)**	**6 (n = 10)**	**7 (n = 15)**	**8 (n = 16)**	**9 (n = 19)**	**10 (n = 10)**	**11 (n = 7)**	**12 (n = 20)**	**13 (n = 20)**
**Duration (s)**	1.74 ± 0.10	1.78 ± 0.13	2.12 ± 0.09	1.98 ± 0.13	1.76 ± 0.08	2.10 ± 0.19	2.31 ± 0.11	2.11 ± 0.08	2.36 ± 0.10	2.13 ± 0.16	1.45 ± 0.20	1.94 ± 0.32	1.91 ± 0.13
**Entropy**	0.64 ± 0.008	0.57 ± 0.02	0.60 ± 0.006	0.64 ± 0.01	0.59 ± 0.01	0.65 ± 0.009	0.61 ± 0.01	0.55 ± 0.01	0.61 ± 0.01	0.58 ± 0.01	0.63 ± 0.009	0.56 ± 0.009	0.54 ± 0.01
**F0 mean (Hz)**	265.2 ± 27.56	171.3 ± 13.4	139.9 ± 4.22	214.9 ± 15.61	243.1 ± 12.93	363.4 ± 27.47	148.9 ± 7.37	183.5 ± 9.90	465.0 ± 34.02	240.6 ± 34.03	494.2 ± 85.58	206.0 ± 7.86	188.8 ± 7.85
**F0 min (Hz)**	70.36 ± 3.39	70.33 ± 2.68	81.96 ± 2.42	68.74 ± 2.65	68.02 ± 2.09	71.92 ± 4.30	72.17 ± 1.82	64.91 ± 0.74	80.33 ± 3.95	74.81 ± 2.26	73.73 ± 8.94	66.17 ± 2.11	70.64 ± 1.58
**F0 max (Hz)**	593.1 ± 72.29	277.5 ± 3.33	175.4 ± 5.37	407.5 ± 38.88	332.9 ± 16.32	714.2 ± 20.39	256.7 ± 13.61	294.2 ± 9.36	866.6 ± 48.39	473.1 ± 76.67	914.5 ± 146.6	284.2 ± 6.91	273.4 ± 8.81
**F0 start (Hz)**	76.41 ± 3.68	71.46 ± 2.73	86.07 ± 1.94	72.85 ± 3.79	69.49 ± 1.77	76.03 ± 4.38	75.55 ± 2.63	65.14 ± 0.79	85.14 ± 3.95	78.98 ± 2.89	80.69 ± 9.22	68.76 ± 1.87	73.96 ± 2.19
**F0 end (Hz)**	160.4 ± 23.55	210.6 ± 22.51	91.90 ± 3.76	251.3 ± 25.70	209.6 ± 27.40	239.2 ± 33.73	133.0 ± 11.90	118.20 ± 18.11	344.1 ± 66.87	148.8 ± 22.73	138.4 ± 24.79	175.6 ± 16.00	206.7 ± 20.31
**Inflex 2**	0.84 ± 0.14	0.49 ± 0.07	0.43 ± 0.02	0.60 ± 0.11	1.16 ± 0.21	0.65 ± 0.09	0.57 ± 0.07	0.48 ± 0.06	0.58 ± 0.09	0.64 ± 0.12	0.75 ± 0.09	0.59 ± 0.07	0.70 ± 0.10
**F0 var (Hz/s)**	554.9 ± 76.53	123.6 ± 11.47	84.53 ± 7.59	296.0 ± 48.45	385.9 ± 36.28	535.2 ± 58.11	144.0 ± 17.20	190.5 ± 8.99	580.8 ± 42.75	361.2 ± 86.79	1177 ± 248.8	198.8 ± 14.75	177.8 ± 15.68
**FM rate (s^{-1})**	5.91 ± 0.65	3.12 ± 0.86	2.71 ± 0.55	3.54 ± 0.57	9.96 ± 0.42	3.72 ± 0.32	2.59 ± 0.44	5.08 ± 0.67	5.46 ± 0.50	5.41 ± 0.77	4.16 ± 0.86	6.02 ± 0.54	5.67 ± 0.45
**FM extent (dB)**	107.8 ± 17.54	69.70 ± 21.30	85.20 ± 34.90	92.57 ± 12.15	38.88 ± 3.48	146.7 ± 14.21	102.6 ± 36.02	48.75 ± 7.08	122.6 ± 13.62	78.53 ± 24.65	298.0 ± 57.1	35.03 ± 2.06	34.94 ± 4.33
**Harmonicity (dB)**	7.93 ± 0.64	11.20 ± 0.66	15.25 ± 1.19	7.87 ± 0.79	3.79 ± 0.69	9.01 ± 0.90	11.16 ± 0.91	14.18 ± 0.72	11.80 ± 0.61	10.26 ± 1.08	8.38 ± 0.77	12.16 ± 0.89	12.53 ± 1.02
**Jitter (%)**	0.04 ± 0.005	0.01 ± 0.002	0.009 ± 0.001	0.03 ± 0.003	0.06 ± 0.005	0.02 ± 0.002	0.02 ± 0.004	0.01 ± 0.0008	0.02 ± 0.002	0.03 ± 0.006	0.05 ± 0.008	0.03 ± 0.003	0.03 ± 0.002

**Shimmer (%)**	0.17 ± 0.008	0.10 ± 0.01	0.06 ± 0.01	0.15 ± 0.01	0.19 ± 0.009	0.15 ± 0.01	0.09 ± 0.009	0.09 ± 0.01	0.12 ± 0.008	0.14 ± 0.16	0.18 ± 0.02	0.11 ± 0.01	0.10 ± 0.009
**AM var (Hz/s)**	48.93 ± 3.85	74.04 ± 3.02	34.42 ± 0.72	50.49 ± 2.38	47.22 ± 1.96	42.75 ± 3.19	36.43 ± 3.06	65.61 ± 1.85	38.32 ± 1.45	65.75 ± 3.28	62.49 ± 6.66	40.09 ± 1.89	43.76 ± 1.81
**AM rate (s^{-1})**	5.76 ± 0.31	7.44 ± 0.54	4.75 ± 0.44	6.34 ± 0.43	5.91 ± 0.46	4.70 ± 0.24	5.18 ± 0.44	6.46 ± 0.57	5.45 ± 0.21	9.26 ± 0.42	4.89 ± 0.62	4.92 ± 0.38	4.20 ± 0.39
**AM extent (dB)**	9.04 ± 1.35	10.44 ± 1.06	8.05 ± 0.77	8.58 ± 0.87	8.59 ± 0.98	9.14 ± 0.51	7.86 ± 0.78	12.30 ± 1.98	7.24 ± 0.41	7.27 ± 0.51	14.60 ± 3.23	9.71 ± 1.25	13.30 ± 1.83
**Deterministic chaos (% of call duration)**	24.62 ± 5.63	7.76 ± 5.79	0 ± 0	32.63 ± 2.97	61.64 ± 5.48	3.87 ± 2.06	4.54 ± 3.73	6.65 ± 2.18	18.54 ± 4.62	8.63 ± 3.74	27.44 ± 5.58	29.56 ± 6.58	24.51 ± 4.78
**Subharmonics (% of call duration)**	0 ± 0	1.54 ± 1.54	0 ± 0	2.44 ± 2.44	4.98 ± 4.98	0 ± 0	0.86 ± 0.59	2.45 ± 1.36	5.39 ± 2.42	9.92 ± 6.89	5.28 ± 5.28	7.50 ± 3.22	1.39 ± 0.98
**Biphonation sidebands (% of call duration)**	12.82 ± 4.87	0 ± 0	0 ± 0	0 ± 0	0.59 ± 0.59	0 ± 0	0 ± 0	1.97 ± 1.97	7.92 ± 3.15	24.75 ± 5.09	21.44 ± 8.37	0.90 ± 0.90	2.77 ± 2.77
**Frequency jumps (frequency)**	2.1 ± 0.31	0.5 ± 0.38	0 ± 0	0.25 ± 0.18	0.2 ± 0.13	0.9 ± 0.28	0.6 ± 0.19	0.75 ± 0.34	2.21 ± 0.15	1.3 ± 0.3	2.14 ± 0.71	0.2 ± 0.12	1.4 ± 0.25

Table S2. Descriptive statistics (mean ± SE) for each of the 21 vocal parameters of a given heifer during the putatively negative contexts (n = 163).

Putatively negatively valenced Vocal parameter	Heifer 1 (n = 30)	2 (n = 10)	3 (n = 3)	4 (n = 7)	5 (n = 2)	6 (n = 7)	7 (n = 21)	8 (n = 24)	9 (n = 5)	10 (n = 16)	11 (n = 2)	12 (n = 10)	13 (n = 26)
**Duration (s)**	1.77 ± 0.05	2.00 ± 0.15	1.66 ± 0.12	2.16 ± 0.28	1.92 ± 0.23	2.71 ± 0.28	2.04 ± 0.10	2.37 ± 0.09	2.12 ± 0.17	2.00 ± 0.11	2.00 ± 0.11	1.94 ± 0.11	1.95 ± 0.10
**Entropy**	0.62 ± 0.007	0.58 ± 0.02	0.60 ± 0.003	0.62 ± 0.01	0.61 ± 0.03	0.65 ± 0.02	0.58 ± 0.04	0.60 ± 0.009	0.60 ± 0.03	0.55 ± 0.01	0.55 ± 0.05	0.60 ± 0.01	0.55 ± 0.007
**F0 mean (Hz)**	259.7 ± 13.81	159.0 ± 11.04	158.7 ± 5.81	214.5 ± 23.96	197.9 ± 73.74	286.0 ± 40.19	157.2 ± 8.53	167.5 ± 9.36	461.8 ± 90.08	280.2 ± 26.45	186.7 ± 33.89	202.9 ± 12.75	163.2 ± 4.92
**F0 min (Hz)**	70.33 ± 2.70	71.69 ± 1.54	96.36 ± 8.06	69.94 ± 2.87	63.17 ± 5.46	65.13 ± 4.49	65.37 ± 1.95	64.25 ± 1.44	93.54 ± 13.90	76.32 ± 3.57	67.98 ± 2.96	74.14 ± 3.74	68.31 ± 1.43
**F0 max (Hz)**	492.5 ± 33.0	257.1 ± 15.83	184.7 ± 3.51	427.1 ± 46.09	254.3 ± 94.84	635.8 ± 37.23	252.7 ± 15.16	309.2 ± 12.98	774.8 ± 109.7	559.3 ± 65.54	353.5 ± 126.8	287.5 ± 16.45	268.5 ± 7.36
**F0 start (Hz)**	71.63 ± 2.76	76.51 ± 3.60	99.97 ± 7.91	73.13 ± 3.41	63.56 ± 5.38	69.58 ± 5.47	75.02 ± 4.63	65.91 ± 1.46	98.30 ± 12.45	80.72 ± 3.10	77.04 ± 3.51	80.43 ± 5.25	72.04 ± 1.52
**F0 end (Hz)**	226.7 ± 11.97	205.6 ± 13.32	105.3 ± 6.09	214.6 ± 29.66	175.4 ± 103.6	280.9 ± 28.57	104.8 ± 13.11	112.9 ± 12.06	261.2 ± 24.24	206.9 ± 37.68	88.88 ± 14.92	142.1 ± 22.66	177.3 ± 17.80
**Inflex 2**	0.54 ± 0.04	0.63 ± 0.09	0.53 ± 0.03	0.87 ± 0.17	1.30 ± 0.78	0.55 ± 0.13	0.51 ± 0.05	0.44 ± 0.03	0.35 ± 0.09	0.76 ± 0.09	0.88 ± 0.40	0.48 ± 0.03	0.68 ± 0.08
**F0 var (Hz/s)**	407.3 ± 37.41	153.9 ± 28.11	100.2 ± 6.51	317.3 ± 56.88	293.4 ± 141.6	344.1 ± 39.09	187.7 ± 18.40	199.3 ± 15.62	556.0 ± 122.2	407.8 ± 57.40	287.1 ± 138.2	239.5 ± 28.54	173.2 ± 12.69
**FM rate (s^{-1})**	6.19 ± 0.23	4.98 ± 0.58	3.58 ± 1.35	5.10 ± 0.76	9.61 ± 0.65	3.79 ± 0.50	5.24 ± 0.46	4.19 ± 0.44	5.15 ± 1.66	4.80 ± 0.43	4.89 ± 1.32	6.39 ± 0.83	5.13 ± 0.32
**FM extent (dB)**	70.50 ± 8.12	33.44 ± 5.34	49.33 ± 28.35	73.42 ± 15.77	29.67 ± 12.72	107.7 ± 25.63	38.16 ± 3.72	57.25 ± 5.84	129.8 ± 20.67	84.00 ± 10.56	71.59 ± 47.59	41.56 ± 5.76	36.62 ± 3.40
**Harmonicity (dB)**	7.72 ± 0.41	8.21 ± 1.46	15.09 ± 1.00	7.92 ± 1.17	4.96 ± 1.62	10.16 ± 1.28	9.37 ± 0.70	11.97 ± 0.49	8.89 ± 1.77	11.60 ± 0.96	11.80 ± 4.61	8.60 ± 0.96	8.77 ± 0.60
**Jitter (%)**	0.04 ± 0.002	0.03 ± 0.006	0.01 ± 0.0008	0.03 ± 0.006	0.05 ± 0.02	0.02 ± 0.003	0.03 ± 0.004	0.02 ± 0.002	0.03 ± 0.008	0.02 ± 0.003	0.02 ± 0.003	0.03 ± 0.004	0.03 ± 0.002
**Shimmer (%)**	0.17 ± 0.006	0.13 ± 0.02	0.05 ± 0.006	0.16 ± 0.01	0.18 ± 0.03	0.14 ± 0.02	0.12 ± 0.01	0.13 ± 0.007	0.12 ± 0.02	0.14 ± 0.01	0.12 ± 0.07	0.14 ± 0.01	0.13 ± 0.007
**AM var (Hz/s)**	52.56 ± 1.30	66.82 ± 3.35	35.94 ± 3.17	48.62 ± 5.04	43.32 ± 5.10	38.39 ± 3.31	40.21 ± 1.59	61.50 ± 2.66	38.20 ± 3.19	70.30 ± 3.16	40.08 ± 4.07	38.88 ± 2.26	43.09 ± 1.53

**AM rate (s^{-1})**	5.38 ± 0.18	9.47 ± 0.85	5.21 ± 0.20	6.36 ± 0.54	7.14 ± 0.56	5.94 ± 0.59	4.74 ± 0.30	7.64 ± 0.30	6.16 ± 0.75	8.61 ± 0.62	4.15 ± 1.80	5.97 ± 0.39	4.58 ± 0.22
**AM extent (dB)**	10.18 ± 0.50	7.78 ± 1.05	6.96 ± 0.85	8.02 ± 0.98	6.05 ± 0.24	7.26 ± 1.51	9.28 ± 0.75	8.48 ± 0.65	6.51 ± 0.84	8.80 ± 0.76	12.41 ± 6.36	6.93 ± 0.73	10.17 ± 0.72
**Deterministic chaos (% of call duration)**	28.64 ± 3.47	22.29 ± 6.70	0 ± 0	19.73 ± 6.56	63.26 ± 10.70	1.34 ± 1.34	25.45 ± 4.56	16.40 ± 4.13	26.94 ± 10.59	6.07 ± 3.05	37.98 ± 13.34	42.56 ± 10.46	29.31 ± 2.34
**Subharmonics (% of call duration)**	4.59 ± 1.56	1.69 ± 1.69	0 ± 0	0 ± 0	0 ± 0	0 ± 0	3.56 ± 1.64	3.99 ± 1.71	1.90 ± 1.90	1.21 ± 1.21	0 ± 0	0 ± 0	3.02 ± 1.15
**Biphonation sidebands (% of call duration)**	22.28 ± 3.86	13.98 ± 6.44	0 ± 0	1.91 ± 1.91	0 ± 0	0 ± 0	0 ± 0	8.08 ± 2.90	19.15 ± 11.88	25.62 ± 5.17	0 ± 0	0 ± 0	3.53 ± 2.12
**Frequency jumps (frequency)**	1.37 ± 0.2	0.1 ± 0.1	0 ± 0	2.0 ± 0.44	1.00 ± 1.00	1.14 ± 0.34	1.00 ± 0.26	1.38 ± 0.23	0.8 ± 0.37	1.38 ± 0.26	1.50 ± 1.50	0.40 ± 0.70	1.23 ± 0.19

Table S3. Tests for the canonical discriminant functions established to discriminate between individual heifers using (A) the putatively positive 'training set' and (B) the putatively negative 'training set'.

	**Function**	**Eigenvalue**	**Variance explained (%)**	**Test of function**	**Wilks' λ**	**χ2**	**df**	**P – value**
**(A)**	1	5.834	41.9	1 – 10	0.002	998.873	120	< 0.001
	2	4.208	30.2	2 – 10	0.012	696.162	99	< 0.001
	3	1.536	11.0	3 – 10	0.063	436.248	80	< 0.001
	4	0.898	6.5	4 – 10	0.159	289.651	63	< 0.001
	5	0.664	4.8	5 – 10	0.302	188.714	48	< 0.001
	6	0.397	2.9	6 – 10	0.502	108.521	35	< 0.001
	7	0.206	1.5	7 – 10	0.702	55.825	24	< 0.001
	8	0.101	0.7	8 – 10	0.846	26.306	15	0.035
	9	0.057	0.4	9 – 10	0.931	11.212	8	0.190
	10	0.016	0.1	10	0.984	2.521	3	0.471
**(B)**	1	6.388	59.1	1 – 9	0.008	737.336	108	< 0.001
	2	2.211	20.5	2 – 9	0.056	435.359	88	< 0.001
	3	1.051	9.7	3 – 9	0.180	259.185	70	< 0.001
	4	0.516	4.8	4 – 9	0.369	150.737	54	< 0.001
	5	0.316	2.9	5 – 9	0.559	87.890	40	< 0.001
	6	0.175	1.6	6 – 9	0.735	46.415	28	0.016
	7	0.103	1.0	7 – 9	0.864	22.019	18	0.231
	8	0.030	0.3	8 – 9	0.953	7.217	10	0.705
	9	0.018	0.2	9	0.982	2.688	4	0.611
